# Study on the Correlation between Humidity and Material Strains in Separable Micro Humidity Sensor Design

**DOI:** 10.3390/s17051066

**Published:** 2017-05-08

**Authors:** Chih-Yuan Chang

**Affiliations:** Department of Civil Engineering, Feng Chia University, No. 100, Wenhwa Rd., Seatwen, Taichung 40724, Taiwan; rchang@fcu.edu.tw; Tel.: +886-424517250 (ext. 3130); Fax: +886-424516982

**Keywords:** humidity sensor, microminiaturization, smart external wall, tile, strain gauge

## Abstract

Incidents of injuries caused by tiles falling from building exterior walls are frequently reported in Taiwan. Humidity is an influential factor in tile deterioration but it is more difficult to measure the humidity inside a building structure than the humidity in an indoor environment. Therefore, a separable microsensor was developed in this study to measure the humidity of the cement mortar layer with a thickness of 1.5–2 cm inside the external wall of a building. 3D printing technology is used to produce an encapsulation box that can protect the sensor from damage caused by the concrete and cement mortar. The sensor is proven in this study to be capable of measuring temperature and humidity simultaneously and the measurement results are then used to analyze the influence of humidity on external wall tile deterioration.

## 1. Introduction

Ceramic tiles are decoration materials commonly used on the external walls of buildings in Asia. They are subject to deterioration, loosening or falling caused by environmental factors such as humidity, temperature, sunlight exposure, acid rain, vibration or earthquakes. In recent years, there have been frequent incidents of injuries caused by falling tiles, the government has implemented legislation requiring that old buildings be regularly examined [1,2,3]. This is a labor intensive task and the safety of buildings with tiles on their external walls could be better ensured with the development of smart and self-diagnosing external walls that use different kinds of sensors to monitor and control signs of tile deterioration. To achieve this goal, the first key step is to be able to measure the temperature and humidity of the external walls.

From the perspective of building medicine [4], the external walls of a building are equivalent to the human skin. Without good care and maintenance, they both are prone to aging or even diseases. In human skin, there is a system of nervous sensors transmitting signals from environmental perceptions. By the same token, buildings require a network of sensors within their external walls to perceive and transmit information about the physical properties of the building structure and the surrounding environment. In addition, after the completion of their construction, the exterior walls of buildings will have to endure severe weathering, including damage caused by wind, rain, humidity and pollution. Among the weathering factors, humidity is one that cannot be ignored [5].

Along with technological advancements, many types of sensors have been used in construction engineering to provide a wide variety of data for management and maintenance. Take RH/T sensors (the sensors used in this study can simultaneously measure temperature and humidity, hence hereinafter they are referred to as RH/T sensors) for example. Currently, they are mostly applied either to monitor indoor environments in order to enable comfortable environment management or to monitor temperature/humidity changes in building structures in order to assess their structural health. This study is unique in its development of a separable RH/T microsensor particularly suitable for very limited and narrow measurement spaces. The sensor developed in this study is protected in a 3D-printed encapsulation and deployed in a cement mortar layer of 1.5–2 cm thickness inside the building’s exterior wall. An experiment was conducted to test if the sensor could measure the temperature and humidity properly and the measurement results were then used to analyze the influence of humidity on the strain of external wall tile deterioration. It is hoped that the sensor developed in this study will become the technological foundation for the research on wear characteristics causing external wall tile deterioration and for the development of IoT-based smart walls or buildings that are capable of self-diagnosing to ensure better public safety.

## 2. Background Research

### 2.1. Application of RH/T Sensors in the Construction Industry

Persistent humidity in a 80~90% range will cause concrete to deteriorate and shorten the life of building structures [6]. Excessively high humidity inside the concrete structure will also promote rusting of rebar, causing the concrete cover to fall off and consequently impairing the structural safety [7]. Therefore, humidity is an indispensable parameter in the monitoring of concrete structures. Currently, research on the application of temperature/humidity sensors (RH/T sensors) in the construction industry can be divided into two types: sensors applied to measure humidity in indoor environments and sensors applied to measure humidity within building structures. There is a large quantity of research on how to use sensors to measure and monitor humidity in indoor environments. For example, energy management platforms have been developed with sensors deployed in indoor environments to have long-term monitoring of the wall surface temperature, relative humidity, sunlight exposure, and CO/CO_2_ concentration in the environment and the monitoring data can provide references for indoor environment management [8]. The monitoring data of the sensors can be used as references to create a comfortable indoor environment through adjustments of heating, ventilation and air conditioning (HVAC) [9]. These sensors have also be used to provide references for the conservation of ancient mosque buildings [10].

For the measurement of humidity inside building structures, some researchers developed new sensing device (5 cm × 4.8 cm × 5 cm) using radio frequency integrated circuits (RFICs) and RH/T sensors [11]. Other researchers developed a 5.2 cm × 4.6 cm × 4.6 cm Smart Temperature Information Material (STIM), which can automatically and continuously transmit concrete humidity data to a Building Physiology Information System (BPIS) for a long period of time [12]. STIM was used in a one-year study comparing the performance of different roof insulation materials for reinforced concrete buildings [13]. Different RH/T sensors were tested with their probes protected by polypropylene encapsulation to find out which sensor could successfully overcome the high alkaline environment found in concrete and continuously monitor the temperature and humidity for two months [14]. Encapsulation boxes (6.5 cm × 4 cm × 6.5 cm) were designed, developed and used to help RH/T sensors overcome the strong alkalinity in the concrete environment [15].

Moreover, some studies also embedded temperature, humidity, and ultrasonic sensors through Wireless Sensor Networks into a precast concrete slab for quality management of the mass and compressive strength of concrete during the curing process [16]. Humidity and temperature are two of the factors affecting the surface corrosion and durability of concrete. Therefore, a study embedded and placed temperature-humidity sensors into concrete blocks in a moist tropical environment. Through a one-year experiment, it became apparent that the west side of a concrete block was most likely to experience carbonation in a high humidity environment [17]. Humidity is very important in the maintenance of concrete structures. If the humidity is abnormal, cracks are easily generated in the concrete structure, causing structural defects. Therefore, a study embedded the humidity sensors into normal-strength and high-strength concretes structures that had just been completely poured, in order to understand the difference in internal relative humidity changes inside concrete structures of different strengths during the early stage of consolidations, and to provide a benchmark as a reference for the maintenance of high-strength concrete [18]. In addition, another study installed displacement sensors into high-strength concrete structures to understand the changes in structural strains of different relative humidity structures through embedded temperature-humidity sensors. The experimental results of this study show that a drier structure had smaller internal strains [19]. Furthermore, a study embedded a humidity sensor into the concrete structure under simulated environment scenario experiments in the laboratory, to understand the impact of different climates on the internal relative humidity and distribution state of the concrete structure. As indicated by the experimental results, among the impact factors of rainfall on structure, the rainfall duration, and whether concrete is exposed directly to the rainwater have greater impact on the internal relative humidity of a structure than the amount of rainfall [20]. The humidity experiments above focus on the concrete’s quality management, durability, crack effects, strain amount, and rainfall effects, respectively. It can clearly be seen that the measurement of humidity is important in concrete structure evaluation. Regarding the strain amount, although the humidity is directly proportional to the strain amount, its more accurate quantification differences are correlated with other deterioration factors, such as tile hollowing rate, which is still pending further study.

There are several cases of RH/T sensors applied to monitor building structures such as a water-proof system on the roof of a museum to make sure the system was functioning properly after it was repaired [21]. RH/T sensors are used in structure simulation specimens together with vibration sensors and network technologies to measure the linear change of stress inside the structure and calculate the correlation between strain and humidity [22].

Even though there have been breakthroughs in solving the problem of the strong alkalinity of freshly mixed concrete, signal transmission and power supply for RH/T sensors with all their electronic circuits, wireless/wired transmission modules and power supply units, they are often quite large in size. Such large sensors are difficult to deploy in the narrow or limited measurement spaces inside building structures, such as the cement mortar layer (often only 1.5–2 cm thick) between the exterior tiles and the reinforced concrete structure of a building, making the development of RH/T microsensors with suitable encapsulation protection to obtain continuous and long-term RH/T measurement data an interesting challenge.

### 2.2. Existing Diagnosis Methods for External Wall Tiles

Like humans, buildings are subject to aging and “death”. So are a building’s external walls. The adherence of tiles to building exterior is a complicated process and each layer of the structure contracts or expand differently when humidity changes, causing the tiles to deteriorate and separate from the building exterior [23]. By collating the literature related to the deterioration of tile external walls, the results show that humidity is also one of the factors that affect environmental deterioration [24]. Moreover, some studies also conducted status surveys and literature compilation, and learned that the impact factors of degradation in tiles of external walls include tile degradation form, degradation range, degradation position, temperature, and humidity [25]. Another study also pointed out that tiles of external walls will experience different levels of spalling, swelling, or shedding, and other deterioration phenomena according to the wind, rain, sunlight, dew, temperature, humidity, sunshine volume, construction quality, and maintenance quality [26]. In addition to environmental factors, there is a study exploring the impact of temperature on the strength of adhesives during the binding process; the results show that when coating a layer of adhesive gel after applying the cement and mortar, if the pasting is not carried out immediately, it will be likely to lead to lower binding strength, and temperature has a significant impact on the strength of adhesives [27]. Even though temperature and humidity are theoretically established by many studies as important factors for the deterioration of building exterior decoration materials (such as tiles), there have been relatively few studies analyzing through long-term monitoring the correlation between humidity and deterioration of external wall decoration materials. It is mainly because of the difficulties in measuring humidity inside concrete or building structures. Measuring the humidity in the interface layer of cement and mortar in a limited space is difficult.

Currently, the most common methods to diagnose external wall tile deterioration are still the visual evaluation method and the tap tone method [28]. The tap tone method is believed to be more economical [29]. Robots can be used for the tap tone method and the diagnosis data are sent to mobile devices through wireless transmission [30]. For external wall decoration materials on high-rising buildings, there are other methods such as the passive quantitative infrared thermography (PQIRT) and errors in the diagnosis data can be corrected through algorithms and experiments [31]. A combination of different methods such as infrared diagnosis, acoustic emission and ultrasound scanning can be used to diagnose the external wall structure and predict its deterioration process [32]. There are also studies that apply acoustic frequency characteristics to test the adherence integrity of tiles [33] or pulse thermal imaging to diagnose the deterioration of tiled building exterior [34].

There is an insufficiency of research on the correlations among factors of external wall deterioration or on the pathological characteristics of the deterioration process. This is probably because the technology for application of humidity sensors inside building external walls is still in an incipient stage. The cement mortar layer exists between the external wall tiles and the concrete structure of a building. Theoretically, when humidity changes, the cement mortar layer and the reinforced concrete will have different levels of contraction or strain. Therefore, in the analysis of tile deterioration factors, it is theoretically necessary to consider the influence of humidity. Nevertheless, among the humidity sensors currently available on the market it is difficult to find ones of the right size for the thin cement mortar layer. Another challenge that needs to be addressed is finding the encapsulation design that can protect the sensors from the highly alkaline concrete environment. As a result, it is a relatively feasible option to develop self-designed sensors of the required sizes and features like the separable RH/T microsensor developed in this study until there are suitable options available on the market.

## 3. Materials and Methods

### 3.1. Humidity Sensor

**Figure 1:**
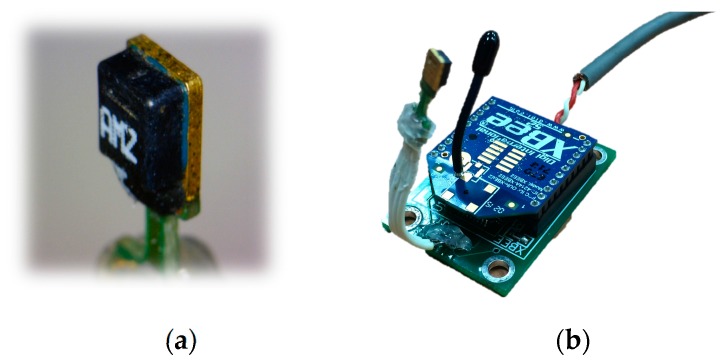
RH/T microsensor developed in this study. (**a**) SHT-series (1×/7×); (**b**) XBee series 2.

The sensor developed in this study is composed of two parts (Figure 1). The first part is a sensing microchip using a SHT-series (1×/7×) digital RH/T sensor with a sensing probe (5 mm × 4 mm × 2 mm) (Figure 1a). The total dimension of the first part is 13 mm × 6 mm × 2 mm. The SHT 1×/7× chip can transmit a calibrated digital output signal. Band gap materials and a 14-digit A/D converter are used in the first part to avoid the need for an extra digital-analog converter circuit. The second part is a wireless signal sending module, which consists of a circuit board, a microcontroller and a wireless transmission module. Equipped with an XBee series 2 chip of the IEEE 802.15.4 stack (Figure 1b), XBee series 2 is an electronic component that is already on the market. This component is currently widely used in low-cost wireless sensor design; the wireless transmission module uses simple serial commands to implement IEEE 802.15.4 ZigBee communication protocols. It also supports three modes: coordinator, router, and end device as well as three network typologies: peer to peer, point to point, and mesh. It operates at the ISM 2.4 GHz band with a maximum transmission distance of 100 m and a maximum transmission speed at 250 kbps. It is often used in wireless sensing networks that are low in cost and power consumption.

When the RH/T sensor was completed, the RH/T sensor was calibrated against the calibrated hand-held hygrometer before the experiment, and the measurement errors of RH/T sensor will be processed via the error calibration programming code scripted in the Silicon Laboratories IDE program; each set of data in every measurement will be processed by the Silicon Laboratories IDE program for error calibration. The RH/T sensor has two means of power supply. The first type uses the CR2450 (3 V) mercury battery for short-term power supply, however, it must be encapsulated to avoid the battery getting damp, and a work space must be set aside for battery replacement. The second type of power supply uses a 5 V/1 A power cable. As there is a requirement for long-term and continuous monitoring, it is recommended that the second type of power supply is used. The power consumption of RH/T sensors depends on the frequency of data capture, taking the first type of CR2450 battery with a nameplate capacity of 660 mA·h, if the frequency of data capture is 30 min, theoretically the power supply life of the battery can reach 10 years.

### 3.2. Strain Gauge

**Figure 2:**
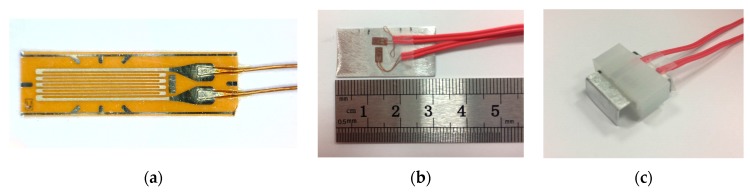
Strain gauge and 3D-printed tunnel-shaped protection device, (**a**) strain gauge; (**b**) pasting design for temperature error calibration; (**c**) tunneling encapsulation.

Strain gauges are commonly used in engineering to measure strain levels. The basic structure of a strain gauge is a foil grid of constant electrical resistance. When in use, it is adhered to the surface of a test object. If the strain of the object changes, the electrical resistance of the foil grid will change accordingly. Any small change in the strain level can be detected and calculated by measuring changes in the electrical resistance (see Figure 2a). Because metal contracts or expands when the temperature changes, another strain gauge of the same specification is often also adhered to the surface of the test object for temperature difference correction (see Figure 2b). The strain gauge was used in this study to measure the strain between the tile adhesion layer (a high polymer mortar layer) and the cement mortar layer. The two ends of the strain gauge were respectively embedded into the high polymer mortar layer and the cement mortar layer. To ensure the aluminum foil of the strain gauge can be extended freely without compromising its structure, 3D printing technology was used in this study to produce a tunnel-shaped protection device (see Figure 2c) to prevent compressional distortion caused by the adhesiveness of the high polymer mortar or the cement mortar layer.

### 3.3. Experiment Design

**Figure 3:**
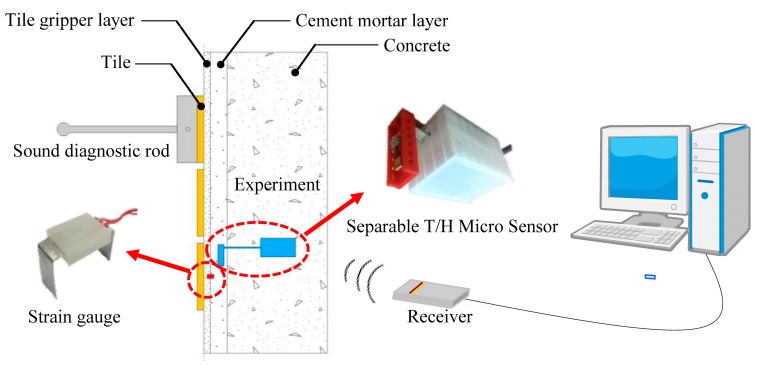
Design concept and structure of the external wall simulation specimen.

The experiments in this study have three objectives. The first objective is to verify if the separable RH/T micro sensors developed in this study can function effectively. The second one is to verify if the 3D-printed encapsulation can effectively protect the front-end sensing chip from potential damage caused by the concrete environment. The third one is to verify if the sensors can simultaneously measure the strain of test objects under different humidity conditions. There were totally two external wall simulation specimens (40 cm × 25 cm × 15 cm each) in the experiment of this study and the strain gage and the separable RH/T micro sensor were embedded in each of the specimens. The strain gage was embedded across the tile adhesion layer (high polymer mortar layer) and the cement mortar layer. The “front-end sensing chip” and “back-end transmission module” of the sensor were embedded respectively into the cement mortar layer and the concrete layer. When the tapping test (external force factor) was conducted on the external wall tiles, the strain level, air temperature/humidity, and temperature/humidity of the cement mortar layers in the specimens (measured by the sensors) were all simultaneously measured. In terms of the signal transmission and reception, the RH/T signals measured by the sensor were first converted into digital signals by the A/D converter inside the sensor. The digital signals were then read, encoded and specified with transmission position by the microcontroller. The encoded signals were then sent to the receiving end via XBee transmission. At the receiving end, the microcontroller decoded the signals and then transmitted the data to the computer end via the UART protocol. The design concept of the experiment specimen and its component is illustrated in Figure 3.

In the evaluation of the strain and hollowing deterioration of the external wall tiles, a method developed by the research team of this study was used. This method was already published and has received several awards. It was developed to evaluate the influence of vibration caused by factors such as earthquakes on the hollowing deterioration of tiles at different humidity levels. In this method, a self-designed tapping device was used to tap on tiles while a directional condenser microphone was used to collect sound samples. Then audio analysis software was used to compare the acoustic energy curves of normal tones and heterogeneous tones. This method can provide quantitative and figure-based evaluation of hollowing deterioration of tiles [35]. In a follow-up study, three external wall specimens decorated with standard glazed tiles (23 cm × 5.5 cm each) were used to simulate external wall specimens with hollowing rates respectively at 25%, 50% and 75%. The tapping device was used on the three specimens and a directional condenser microphone was used to collect sound samples. The method of acoustic energy analysis was used to verify the characteristics of the normal and heterogeneous tones and, consequently, develop a feasible model of acoustic diagnosis for external walls of buildings [36]. In another study, the design of the tapping device was optimized and used on three external wall simulation specimens with hollowing rates respectively at 0%, 30% and 70%. The sound samples were collected and analyzed using acoustic analysis software to improve the scientific method of external wall tiles diagnosis [37].

In summary, this study conducted the impact-echoing experiment using the external wall tiles diagnosis technology in the existing study. The experiment can roughly be divided into five steps:

*Step 1*:  The angle between the specimen and the ground of the experimental site is adjusted to 90° through a laser level, unified specimen positioning can ensure consistency during the hitting of each specimen.
*Step 2*:  To avoid the hitting position being different before and after, the hitting position is marked on the preset tiles to be hit.
*Step 3*:  To maintain the consistency of collected sound samples, the distance between the directional microphone and the specimen is unified to be 30 cm.
*Step 4*:  The angle between the tapping device and specimen surface is adjusted to be 90°, so that the tapping device is hitting the tiles in a free falling manner, while the directional microphone is used to collect the sound samples.
*Step 5*:  The eigenvalues of sound samples are analyzed using the sound analysis program, WaveSurfer.

**Figure 4:**
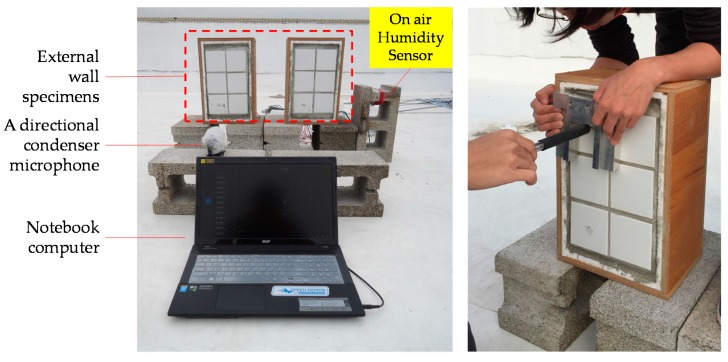
Experiment device (**left**) and tapping method (**right**).

In this study, the self-designed tapping device was used to tap on the tiles of each specimen for a test cycle of 200 taps by rotation to simulate the influence of external factors (see the right of Figure 4). The simulation period was totally four working days until the tiles of the specimen were damaged (at a hollowing rate of 100%). During the tapping process, a directional condenser microphone was used to record the sound samples and the acoustic features of the samples were analyzed to find out the correlation between the accumulation of tapping force and the delamination rate of the tiles at different humidity levels. The device and site arrangement in the experiment of this study are illustrated in the left part of Figure 4.

**Figure 5:**
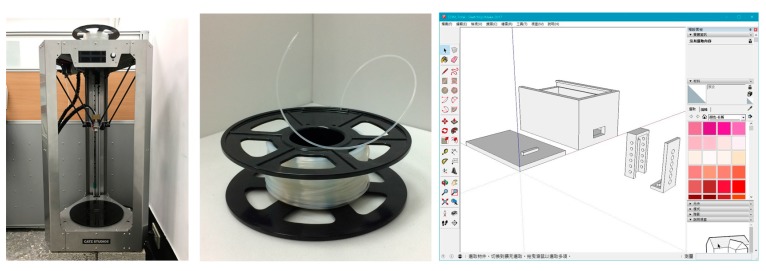
3D printer, material and software.

The 3D printer used in this study was a DIY 3D printer (45 cm × 40 cm × 90 cm) with a maximum nozzle precision of 0.04 mm (see the left of Figure 5), capable of printing out a cylinder of 24 cm in diameter and 36 cm in height at maximum. In the selection of 3D printing material, even though metallic materials can give better strength to the encapsulation box, such materials can also easily interfere with wireless signal transmission and, consequently, affect the accuracy of RH/T measurement. Therefore, polylactic acid (PLA) was selected as the 3D printing material in this study to print out the encapsulation boxes that could protect the electronic components without compromising the measurement accuracy. Also known as corn starch resin, PLA is mainly made from fermented and polymerized starch from corn, wheat and other crops. It is pliable, environmentally friendly and economical; therefore, it is a common 3D printing material (see the center of Figure 5). Sketch Up 2016 was used to draw the encapsulation box design (see the right of Figure 5). When the design drawing was completed, the software would automatically show the printing time, length of the printing material to be used and weight of the finished product.

The encapsulation of the separable micro humidity sensor developed in this study is realized using 3D printing technology. In addition to providing certain strength of protection for the electronic components of the sensors, the 3D-printed encapsulation is, most importantly, lower in cost and more pliable. The plastic material used in 3D printing is resilient to the strong alkalinity in newly mixed cement and does not impede radio signal transmission. Therefore, 3D printing technology is very suitable for the encapsulation of sensor electronic components in this study.

## 4. Results and Discussion

### 4.1. Feasibility of the Separable RH/TMicro Sensor

**Figure 6:**
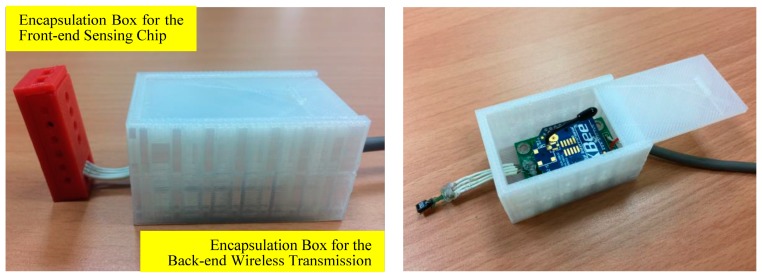
Encapsulation design for the separable RH/T microsensor.

The sensor developed in this study is intended to be embedded into a cement mortar layer with a thickness of just 1.5–2 cm between the tiles and the reinforced concrete structure of a building. The sensors available on the market are not suitable for this task for they are all relatively sizable with the assembly of RH/T sensing chips, circuit boards, microprocessors and wireless transmission modules. Therefore, a separable sensor design was used this study, separating the sensor into two parts: “front-end sensing chip” and “back-end wireless transmission module”. In addition, the strong alkalinity, water, hydration heat, drying shrinkage and compression during the cement pouring process will damage the electronic components and wires of the front-end chip and the back-end module. Therefore, encapsulation protection is needed. In particular, a small measurement space is required for the sensing chip in the encapsulation box instead of completely insulating the sensing chip from the external environment. In this study, 3D printing technology was used to achieve fast and economical fabrication of the encapsulation boxes (see Figure 6).

The encapsulation box for the back-end wireless transmission module was 5.2 cm × 3.5 cm × 2.8 cm in size. To ensure sufficient strength of the box body, the box adopted a multi-layer structure with walls of 3 mm in thickness. The encapsulation box for the front-end sensing chip was 3.2 cm × 1.4 cm × 0.9 cm in size. To ensure effective and accurate measurement of the temperature/humidity in the cement mortar layer, several round-shaped air holes (Φ = 2 mm~3 mm) were added to the encapsulation box without compromising the encapsulation structure. A roll of PLA weighs 1000 g and costs approximately USD $19 (at the exchange rate of 1 USD = 31 Taiwan dollars). The encapsulation box for the front-end sensing chip weighs three g and that for the back-end transmission module weighs 19 g. In other words, the encapsulation cost is USD $0.057 for the front-end chip, USD $0.361 for the back-end module, and totally USD $0.418 for the complete unit.

### 4.2. Signal Reception and Transmission

The encapsulation design for the separable RH/T micro sensor developed in this study can protect the electronic components and wires of the sensor from damage caused by strong alkalinity, water, heat of hydration, drying shrinkage and compression in the concrete environment. The design can also allow the sensor to not only effectively measure the temperature/humidity but also transmit/receive signals. In the experiment of this study, the RH/T measurements by the front-end sensing chip were transmitted via the wireless transmission module embedded in the cement mortar layer once per minute to the receiver outside the external wall simulation specimen. The receiver then stored the received data in the form of Excel file on a remote computer. The front-end sensing chip was embedded in the cement mortar layer of the specimen while the back-end wireless transmission module was intentionally separated and embedded in the concrete layer of the specimen. The signal was transmitted from the inside of the concrete and the effective signal reception range of the receiver was two meters.

**Figure 7:**
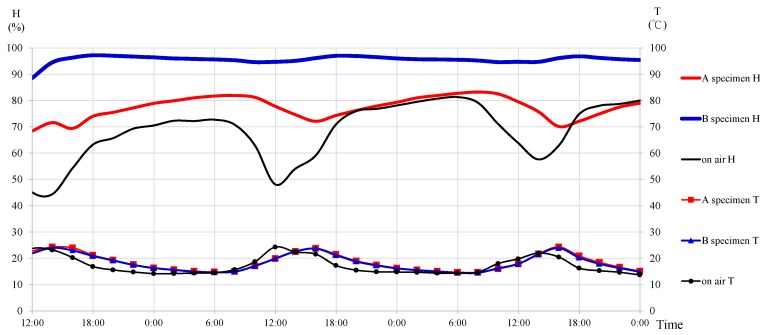
Results of Temperature and Humidity Measurements.

There were three RH/T measurement points in the experiment of this study. The separable RH/T microsensors for the three measurement points were of the same specification. Their probes could simultaneously measure the temperature and humidity. The first measurement point was the cement mortar layer inside Specimen A; the second measurement was the cement mortar layer inside Specimen B; and the third measurement point (On Air) was exposed to the air and deployed next to the two specimens, measuring directly the air temperature and humidity. The temperature and humidity in the air are often negatively correlated with each other. This is also reflected in the measurement results of the On Air point (see Figure 7), which indicate that when the temperature in the air increases, the humidity in the air decreases. 

The humidity at the first measurement point (Specimen A) was between 70% and 80% while that at the second measurement point (Specimen B) was between 90% and 95%. Even though there was still a negative correlation between the humidity and temperature respectively at the first and second measurement points, the correlation was not as significant as that at the On Air point. It was mainly because the first and second measurement points were embedded in the cement mortar layers. Even though the temperature change measured by the front-end sensing chip were consistent with that at the On Air measurement point, the encapsulation box of the sensing chip was only 3.2 cm × 1.4 cm × 0.9 cm and it was difficult to have any drastic change in the humidity level within such a small measurement space. However, the temperature measurement results of Specimens A and B were also synchronous and they both demonstrated a reasonable trend of delayed response to the On Air measurement results. In addition, there was no missing signal reception during the experiment period. Therefore, it could be initially established that the temperature and humidity at the three measurement points could be effectively and continuously measured by the sensor developed in this study.

### 4.3. Influence of Humidity on Tile Deterioration

**Figure 8:**
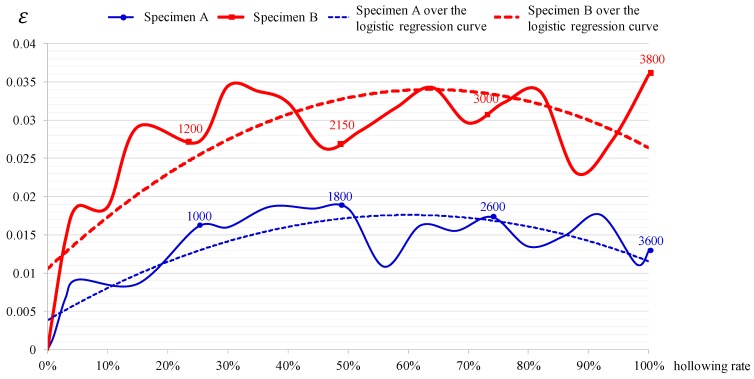
Correlation between humidity and strain (tile deterioration).

Figure 8 lists the results of the experiment on the influence of humidity on tile deterioration (strain) in this study. A negative strain value indicated compression while a positive strain value indicated extension. When the same amount of tapping force was applied on the tiles of the two specimens in this study, the strain values of both specimens were both positive at different humidity levels. The humidity of the cement mortar layer in Specimen A was relatively normal (approximately 70%) and the tiles were broken when the tapping amount reached 3600 and the strain ranged between 0.01 and 0.02. The humidity inside Specimen B was higher (approximately 90%) and the tiles were broken when the tapping amount reached 3800 and the strain ranged between 0.025 and 0.035. The strain of Specimen B with more humidity was significantly higher than that of Specimen A with lower humidity. When there was a 20% difference in humidity, the maximum strain difference could reach approximately 50%. However, this experiment is an initial attempt to explore the influence of humidity on tile deterioration, more measurement samples is needed for further verification.

According to the findings of existing research, once the hollowing rate of external wall tiles exceeds 50%, regular external wall safety inspections are needed. When the hollowing rate reaches 100%, the tiles are broken and fall off the walls. A logistic regression analysis was conducted on the experiment results. As indicated by the analysis results, the explanatory power (R2) of Specimen A over the logistic regression curve was 0.74 and that of Specimen B was 0.7, indicating significantly high accuracy of the experiment results. Based on the experiment results, the equations between humidity and strain in Specimen A Equation (1) and Specimen B Equation (2) were developed, in which the “Y” value refers to the strain and the “X” value is the hollowing rate. The strain values of both specimens peaked when the hollowing rate was approximately 60%. This finding can be used for the future development of smart external walls. When the strain level reaches the peak and then gradually decreases, the smart external walls will automatically alert building managers to inspect or repair the external wall tiles:

y = −0.038x^2^ + 0.0458x + 0.0038 (1)

y = −0.0578x^2^ + 0.0736x + 0.0106 (2)

This research has verified that the separable RH/T microsensor developed in this study can be used to measure the temperature and humidity in the cement mortar layer and to evaluate the correlation between humidity and tile hollowing rate. However, this study is only a small-scale pilot study. More experiments are needed to produce more statistically valid data in order to improve the accuracy of the correlation model and finally develop a feasible diagnosis system that can detect building pathology characteristics.

### 4.4. Discussion

It is a challenging task to embed electronic components in the concrete or cement mortar layer. Problems with issues such as the sensor size, signal transmission, water and strong alkalinity in the environment, and protection of the electronic components must be addressed. In this study, the separable sensor design was used in this study, separating the sensor into the front-end sensing chip and the back-end wireless transmission module. The size of the encapsulation box for the front-end sensing chip was reduced to 3.2 cm × 1.4 cm × 0.9 cm and the chip could successfully measure the temperature/humidity in the 2 cm-thick cement mortar layer. However, there are still many research restrictions and problems that need to be overcome in this study. They are discussed as follows:

(1) *Study limitations*: There were only two specimens in the experiment of this study. The experiment underwent several failures and corrections before it was finally successful. Problems such as signal anomaly, transmission disruption, electronic component damage and insufficient encapsulation protection occurred in the experiment. Due to the constraints in budget and time, the initial successful experiment results are used in the discussion of this study without conducting more experiments. With more human resources and financial resources in the future, more experiments will be needed to provide more data for further analysis.
(2) *Signal transmission distance*: The back-end wireless transmission module was intentionally embedded in the concrete layer and its effective transmission range was approximately two meters. It is suggested that, in the future application of this separable sensor developed in this study, longer connecting wires between the front-end chip and the back-end wireless transmission module should be used so that the back-end module will be moved outside the concrete structure. When there is no barrier, the transmission range of the module in the air will increase to 100 m at most.
(3) *Damage prevention*: Damage to the electronic components and wires caused by the strong alkalinity, water, hydration heat, drying shrinkage and compression during the process of cement pouring can result in loss of partial functions or accuracy of the sensor or even disruption of the experiment. Another option is to embed the electronic components and wires after the cement pouring or the cement mortar layer is completed. However, this option is costlier and difficult to implement along with other difficulties with measurement validity, encapsulation sealing and signal transmission. Deployment of electronic sensing devices either before the pouring of cement or after the competition of the building structure has its own problems to overcome. Selection of which option to use must consider the requirements of each case as well as the technological feasibility, economic benefits and ease of maintenance.
(4) *Improvement of encapsulation*: 3D printing technology was used in this study to provide encapsulation protection for the front-end sensing chip and the back-end wireless transmission module. The internal space of the encapsulation box for the front-end chip was 28 mm × 10 mm × 4.5 mm (1.26 cm^3^ in volume) with several air holes (Φ = 2 mm~3 mm) on its walls. Even though the encapsulation box for the front-end chip was proven feasible, water or strongly alkaline liquid running through the air holes could still easily damage the chip. To solve this problem, improvements can be made with the adjustment of the air hole (such as changing the shape, size, location and orientation of the air hole) or with the use of innovative air hole screening materials (such as breathable fiber fabrics). In this study, the compression on the encapsulation box was not severe. However, it is research-worthy to design an easy-to-repair encapsulation box in the future.

## 5. Conclusions and Suggestions

The goal of this study is to solve the problems of RH/T measurement in limited spaces in concrete structures. A separable RH/T microsensor was developed in this study. The sensor was composed of a front-end sensing chip and a back-end wireless transmission module. 3D printing technology was used to produce encapsulation boxes for the front-end chip, the back-end module and the connecting wire in between, protecting them from damage caused by the compression, strong alkalinity and moisture from the cement mortar and concrete layers. The 3D-printed encapsulation box used in this study at a total cost of USD $0.418 each is very cost-effective. The 3D design software required for this encapsulation box is for free and easily accessible. The form, specification, material and color of the encapsulation box can be easily changed as well. For the research on how to protect electronic components to be embedded in concrete structures, 3D printing is a very useful and economical tool.

Even though it was verified that the separable microsensor developed in this study is capable of continuously measuring the temperature and humidity in the cement mortar layer and its measurement results can be used to analyze the correlation between humidity and tile deterioration, this study is only a small-scale pilot study. More experiments and studies are needed in the future to produce more statistically reliable and valid data in order to improve the accuracy of the correlation model and finally develop a feasible diagnosis system that can detect characteristics of building pathology. In addition, future research is suggested to incorporate the IoT technology with different sensors monitoring the temperature, humidity, strain, and sunlight exposure inside and outside the building. With the development of smart external walls capable of self-diagnosing based on the characteristics of tile deterioration, we will be a step closer to the future of people living in smart buildings that will self-diagnose the deterioration of their external wall tiles and automatically report to the building managers to ensure better public safety management for everyone in the city.
